# Postoperative Pain following Pulpotomy of Primary Molars with Two Biomaterials: A Randomized Split Mouth Clinical Trial 

**DOI:** 10.22037/iej.2017.02

**Published:** 2017

**Authors:** Leili Shafie, Hamide Barghi, Masoud Parirokh, Hamed Ebrahimnejad, Nozar Nakhae, Sara Esmaili

**Affiliations:** a * Pedodontist, Fellowship in Sedation and Hospital Dentistry, Kerman, Iran; *; b * Department of Pediatric Dentistry, Dental School, Shiraz University of Medical Sciences, Shiraz, Iran; *; c * Oral and Dental Diseases Research Center, Dental School, Kerman University of Medical Sciences, Kerman, Iran; *; d * Department of Oral and Maxillofacial Radiology, Dental School, Kerman University of Medical Sciences, Kerman, Iran; *; e * Kerman Neuroscience Research Center ,Kerman ,Iran; *; f *Department of Pediatric Dentistry, Dental School, Shiraz University of Medical Sciences, Shiraz, Iran*

**Keywords:** Calcium-enriched Mixture, Mineral Trioxide Aggregate, Pain Measurement, Primary Molar, Pulp Capping. Pulpotomy

## Abstract

**Introduction:**

The aim of this randomized clinical trial split-mouth study was to compare the postoperative pain following use of mineral trioxide aggregate (MTA) and calcium-enriched mixture (CEM) cement as pulpotomy agents in carious primary molars.

**Methods and Materials:**

Forty-seven children aged between 6-10 years old were enrolled in this study. Each child had two cariously involved primary molar in need of pulpotomy. After caries removal and preparing access cavity in one of the carious teeth, either MTA or CEM cement was randomly used as the pulpotomy agent, while the other cariously involved primary molar tooth was capped with the other material in a separate visit. After covering the radicular pulp with one of the capping materials the teeth were permanently restored with stainless steel crown (SSC). Postoperative pain was recorded by using Wong-Baker faces pain rating scale (Wong-Baker FPRS) up to seven days following the treatment. Data was analyzed using the Wilcoxon, McNemar, and chi square tests.

**Results:**

Forty-five patients fulfilled the treatment procedure and returned the Wong-Baker FPRS forms. Overall 65.6% of the patients reported pain irrespective of the pulpotomy agents used. There was no significant difference in postoperative pain between the teeth that received either MTA or CEM cement as pulpotomy agents in the first, second and the third day (*P*=0.805, *P*=0.942, *P*=0.705, respectively) following the procedure. The trend of the pain scores showed decreasing manner during the study period for the teeth in either groups of MTA or CEM cement. There was no significant difference between the two groups in the number of analgesics used following the treatment (*P*>0.05).

**Conclusion:**

The findings of the present study showed that a majority of the children felt pain following pulpotomy and SSC placement; however, there was no significant difference in pain reported when either MTA or CEM cement was used as pulpotomy agents.

## Introduction

Each year, a large number of primary molars receive pulpotomy treatment because of extensive caries and pulp exposure [1]. Most pediatric dentists believe that pulpotomy is the treatment of choice for vital primary molars compared to the other vital pulp therapy techniques [2]. Pulpotomy is a dental procedure that can be defined as the amputation and removing of coronal part of the pulp tissue followed by covering the radicular pulp tissue with a therapeutic material such as calcium hydroxide (CH), mineral trioxide aggregate (MTA), calcium-enriched mixture (CEM) cement and zinc oxide eugenol (ZOE) [3, 4]. This procedure has been advocated for primary molars as well as permanent teeth with open apices that have either carious or traumatic pulp exposures.

Several systematic reviews and meta-analyses have shown that MTA could be named as one of the most suitable materials for pulpotomy in primary molars because of high clinical and radiographic success rate compared to other pulpotomy agents [5-8]. CEM cement is a newly introduced biomaterial that has shown promising results and comparable clinical and radiographic success rate with MTA [3, 9, 10]. 

One of the important points in performing dental procedures for the children is the amount of pain they may suffer from that whenever the dental pulp has involved. A few investigations regarding prevalence of postoperative pain in pediatric dentistry have been published. The results of these studies have shown that both pulpotomy and stainless steel crown (SSC) placement procedures, are among the most prevalent dental procedures that may cause post-treatment pain in children [11-13]. So far, all studies on vital pulp therapy in primary teeth evaluated the success rate of either the procedure or the capping materials [9, 10, 14, 15]. However, there is no clinical investigation on pediatric dentistry that assessed pain following use of various pulpotomy agents in primary molars. 

Therefore, the aim of the present study was to compare postoperative pain and number of analgesic consumption following use of two different pulpotomy agents namely MTA and CEM cement in primary carious molars.

## Materials and Methods

This split mouth randomized clinical trial study was approved by the Ethics Committee of Kerman University of Medical Sciences in Kerman, Iran (Grant No.: KA/91/267) the procedure was also registered online (Iranian Registry of Clinical Trials ID: IRCT2013091914712N1). Considering *α*=5% and power of 80%, the sample size of 46 patients was calculated.

A total of 47 patients aging between 6 to 10 years old from both genders who referred to the postgraduate clinic of the Pediatric Department of Kerman Dental School in Kerman, Iran from October 2012 to March 2013 could participate in this study.

The patients’ parents were comprehensively informed about the aim and method of the study and an informed consent has been obtained from all of them. 

The inclusion criteria were: 6-10 year-old patients having bilateral primary molars with carious lesion with restorable crown, no tenderness to percussion and no pain except after eating, teeth without clinical and/or radiographic signs or symptoms of pulp degeneration, presence of sinus tract, no physiologic resorption (less than two-thirds of the root length were affected) and presence of active bleeding at the time of the access cavity preparation indicating a vital pulp [16].

Based on the Frankl Behaviour Rating Scale (FBRS) [17], all participants were in grade 3 and 4. The common behavioral control methods such as tell-show-do (TSD) were used.

Exclusion criteria were any systemic diseases that should not receive vital pulp therapy such as leukemia and some types of malignancies, radiographic findings such as internal resorption, widening of periodontal ligament (PDL), furcation and apical radiolucency, and root canal calcifications [16, 18].

Based on a random selection (by using table of random numbers) each tooth randomly received either one of the pulpotomy agents, MTA (Tooth-colored ProRoot Dentsply, Tulsa Dental, Tulsa, OK) or CEM cement (BioniqueDent, Tehran, Iran) in a split mouth design. 

After application of local anesthesia using 2% lidocaine with 1:80000 epinephrine (Darou Pakhsh, Tehran, Iran) and rubber dam isolation, pulpotomy was performed with a round bur (Tizkavan, Tehran, Iran) by a high-speed handpiece with copious water irrigation. Then the rest of coronal pulp was removed by a sharp excavator. After full pulpotomy the cavity was rinsed with 0.9% normal saline and hemostasis was tried to achieve by a wet cotton pellet [19]. If the hemostasis could not be achieved for each one of the treated teeth the patient was excluded from the study and pulpectomy was performed for the tooth. In the teeth with successful radicular pulp hemostasis, either MTA or CEM cement was randomly used as the pulpotomy agent for one tooth, while the other cariously involved primary molar was capped with the other material in a separate visit. After setting of the pulpotomy agent, restorative glass ionomer cement (GC Corporation, Tokyo, Japan) was placed over the pulpotomy agent immediately followed by a final SSC restoration(3M ESPE, Norristown, PA, USA) [19]. 

All dental procedures including anesthesia, pulpotomy and placement of the SSC were performed by a second year postgraduate pediatric dentistry student. Patients’ parents were asked to complete a Wong-Baker faces pain rating scale (Wong- Baker FPRS) questionnaire by asking their children’s pain and rate their pain intensity up to 7 days after the treatment and bring that questionnaire back after seventh day. The Wong-Baker FPRS consists of a row of 6 numbered faces ranging from “no hurt” to “hurts worst” [12, 13].

**Table 1:** Demographic and clinical features of the patients

**Variable**	**CEM/MTA (N)**
**Gender**	
Male	17
Female	28
**Objective **	
No pain	35
Pain upon chewing	10
**Treated teeth**	
Right maxillary first molar	9
Left maxillary first molar	9
Right maxillary second molar	7
Left maxillary second molar	7
Right mandibular first molar	22
Left mandibular first molar	22
Right mandibular second molar	7
Left mandibular second molar	7

**Figure 1:**
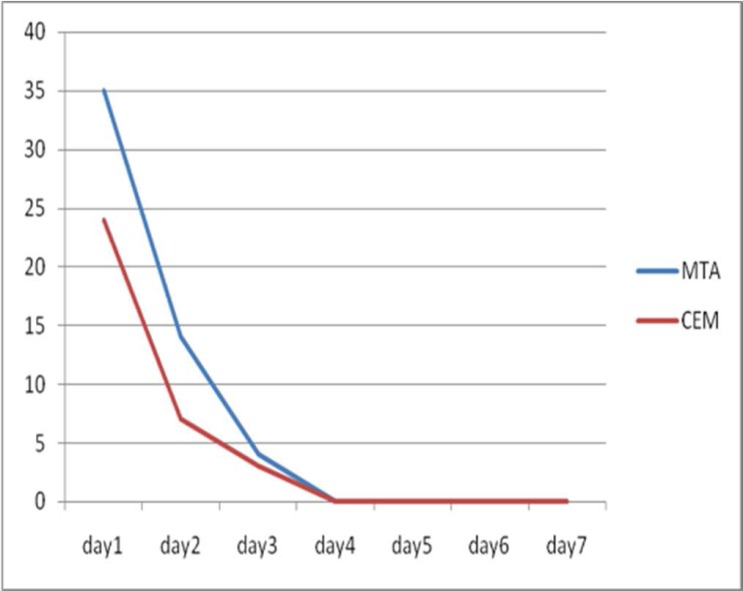
The number of patient who had pain up to day 7 after the treatment

Scoring of pain severity was outlined as follows; 0: no pain, 1-3: mild pain, 4-6: moderate pain and 7-9: severe pain [20]. According to the mean age and weight of studied children, 325 mg analgesic tablets were preferred to syrup. Moreover, for better statistical analysis, tablets are more precisely evaluated than administrated syrup. The parents were supplied by 325 mg acetaminophen tablets (Daruo Pakhsh, Tehran, Iran) as an analgesic in case of pain and were asked to record the number of analgesic used on the patient’s pain questionnaire. The maximum use of analgesic was a tablet every 6 h and the parents were given an emergency phone number if there was any need for consultation in case of severe pain. In order to achieve double blindness, each Wong-Baker FPR scale had a code number to show the pulpotomy agent used. Furthermore, the parents who recorded the pain intensity and number of used analgesics were unaware of the study groups. 

The data were analyzed by using the Wilcoxon, McNemar and chi square tests. The level of significance was set at 0.05.

## Results

From the 47 patients who met the inclusion criteria and their parents agreed to enroll in the study, 2 subjects were excluded because one patient did not return the Wong-Baker PRFS after the second visit and one patient used ibuprofen syrup as an analgesic for pain relief. The rest (45 patients with the mean age of 7.1±1.006 years) completed the forms and returned it to the department. The patients’ demographic data is presented in Table 1. Overall the majority of the patients (65.6%) reported pain following treatment irrespective of the type of pulpotomy agents used. 

**Figure 2:**
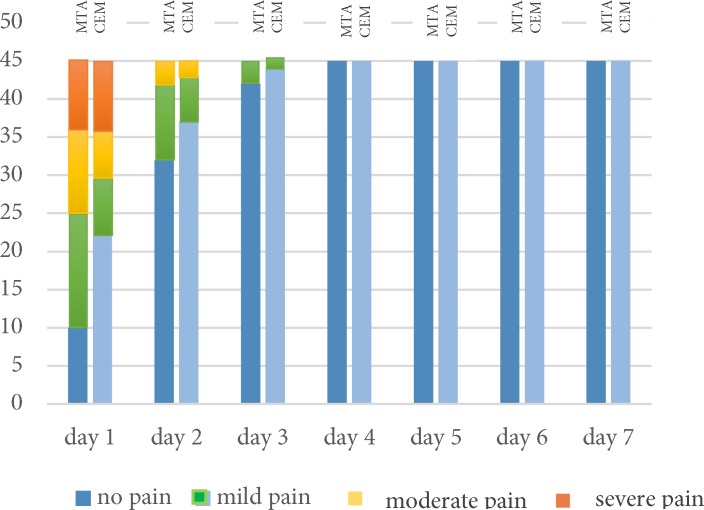
Different levels of pain after pulpotomy

Overall there was no significant difference between pain reported after using CEM cement and MTA as pulpotomy agents (*P*=1.00). Data analysis showed that as time passed, patients reported lower pain rate irrespective of the pulpotomy agent used (Figure 1 and Table 2). Pain trend showed that after the third day none of the patients in either groups reported pain until the end of the study period. Results of the present study showed that only at the first day following the treatment the patients reported sever pain (Figure 2). 

There was a significantly higher analgesic consumption in patients who reported pain compared to the patients that had not pain following the treatment (*P*<0.0001). 

**Figure 3:**
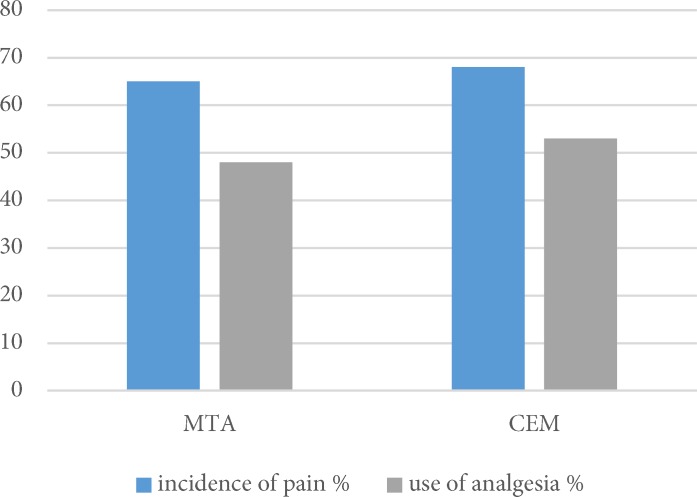
Incidence of pain and use of analgesic in two groups

There was no significant difference between the pulpotomy agents in consumption of analgesics following treatment (*P*>0.05) (Figure 3).

## Discussion

In the resent study no significant difference was found in pain following pulpotomy using two pulpotomy biomaterials including MTA and CEM cement. 

Both American academy of pediatric dentistry (AAPD) and British society of pediatric dentistry (BSPD) recommended placement of the SSC after pulpotomy in primary molars and have described SSC as a durable, relatively inexpensive treatment with minimal technique sensitivity during placement as well as an advantage of full coverage crown. Therefore, in the present study SSC was used as final restoration [21, 22].

Previous investigations have shown that 33 to 95% of children reported pain following dental procedures. These investigations either evaluated postoperative pain in children following dental procedures that had been performed under general anesthesia or local administration of the anesthetic agents [12, 13, 23]. There was no previous investigation that compared postoperative pain with different pulpotomy agents in primary molars. After measuring postoperative pain and discomfort, Staman *et al.* [13] reported that children who received pulpotomy and SSC had significantly higher pain and discomfort compared to other dental procedures. Results of the present study showed that the majority of children (65.6%) had pain following pulpotomy and SSC placement irrespective of the pulpotomy agent used. Ashkenazi *et al.* [11] reported that 56.4% of the children who received pulpotomy with formocresol and SSC reported pain following the treatment that is comparable with the results of the present study indicating 65.6% post-treatment pain following the same procedure.

**Table 2:** Mean (SD) of pain scores at different periods after pulpotomy with CEM/MTA

	**Mean (SD) of pain (CEM/MTA)**	***P*** **-value**
**Day 1**	2.3 (1.1)/2.2 (1.1)	0.805
**Day 2**	1.2 (0.57)/1.2 (0.57)	0.942
**Day 3**	1 (0.25)/1 (0.28)	0.705

No significant relationship was found between the patients’ gender, type of the treated tooth and post-operative pain. This was consistent with another study [24]. Regardless of the pulpotomy agent, the tissue ulceration which happens during the pulpotomy procedure, is the main cause of pain after pulpotomy.

The reason that MTA and CEM cement had been compared in the present study was the successful outcome of vital pulp therapy investigations that used these biomaterials in primary molars [9, 10]. Asgary *et al.* [25] in an investigation compared CEM cement and MTA as pulpotomy agents in cariously exposed permanent teeth and reported no significant difference in patients’ pain following treatment. The results of the present study that used the same pulp capping agents provided the same findings for the primary molars. In the current study, the patients received pulpotomy followed by SSC. Each one of the procedures may result in pain following the treatment. However, according to the previous studies [13], placing SSC might be the major etiologic factor for the post-operative pain. 

In the present study, the trend of pain showed that only during early post-treatment phase the patients experienced pain. 

Therefore, it would be reasonable to give some information to the parents that their children received pulpotomy and SSC treatment regarding a possibility of pain until the third day after the treatment and prescribe on-demand mild analgesic.

Fortunately, severe and moderate pain was reported only at the first and the second day following treatment (Figure 2). The authors recommend whenever pulpotomy and SSC have been performed for a child the practitioners should instruct the parents to monitor their kid and use on-demand analgesics. 

The present study was designed as split-mouth randomized clinical trial because each patient received both pulpotomy agents for different primary molars in need of vital pulp therapy. The advantage of split-mouth randomized clinical trial is the fact that each patient served as his/her own control and the results would be more reliable as individual differences had the least effect on the outcome of the study; however, a new investigation could not find significant differences in intervention effect between parallel-arm randomized clinical trials compared to those with split mouth design [26]. 

Nowadays acetaminophen is the most common analgesic used in pediatrics in the US. Acetaminophen is an effective safe analgesic for mild to moderate pain relief and for these reasons had been prescribed as the analgesic in the present study [27]. The importance of recording the number of analgesic used by the patient is understanding the severity of the patients’ pain [20].

Pain assessment following pulpotomy and SSC placement could be a limitation for our study. In future, it is suggested to accomplish these two treatments separately so that the precise evaluation of post-operative pain could be readily obtained after each step.

## Conclusion

No significant difference was found in pain levels following pulpotomy with either of cements as pulp capping agents in carious primary molars. Meanwhile prescribing analgesic at least for a few days following treatment should be considered.
